# Downregulation of lumican accelerates lung cancer cell invasion through p120 catenin

**DOI:** 10.1038/s41419-017-0212-3

**Published:** 2018-03-16

**Authors:** Cheng-Ta Yang, Jhy-Ming Li, Wing-Keung Chu, Shu-Er Chow

**Affiliations:** 1 Department of Thoracic Medicine, Chang Gung Memorial Hospital, No. 5 Fu-Hsing Street, Guishan District, Taoyuan, Taiwan; 2 Department of Respiratory Therapy, College of Medicine, Chang Gung University, No. 259, Wen-Hwa 1st Road, Guishan District, Taoyuan, Taiwan; 3 Department of Surgery, Division of Colon and Rectal Surgery, Chang Gung Memorial Hospital, No. 6, West Section, Chiapu Road, Putzu City, Chiayi Taiwan; 4 Department of Physiology, College of Medicine, Chang Gung University, No. 259, Wen-Hwa 1st, Guishan District, Taoyuan, Taiwan; 5 Department of Nature Science, Center for General Studies, Chang Gung University, No. 259, Wen-Hwa 1st, Guishan District, Taoyuan, Taiwan; 6 Department of Otolaryngology, Head and Neck Surgery, Chang Gung Memorial Hospital, No. 5 Fu-Hsing Street, Guishan District, Taoyuan, Taiwan

## Abstract

The overexpression of lumican has been found in lung cancer cells; however, the functional role of lumican in lung cancer cells remains unclear. In this study, we found lumican functioned as a tubulin-binding protein and the depletion of lumican by transfection with its specific shRNA increased lung cancer cell invasion. Such alterations led to morphological changes and actin cytoskeleton remodeling, including the induction of membrane ruffling or protrusion and stress fiber formation, correlated with the increased activities of Rac and Rho. The downregulation of lumican was also implicated in macrophage-conditioned media (maCM)-induced cell invasion. Immunofluorescence images and immunoprecipitation assays revealed the co-localization of p120-catenin (p120ctn) and lumican. Reduction in the levels of p120ctn induced membrane ruffling and the activation of the Rho family, which accelerated cell invasion. Our data indicated that lumican is associated with microtubule-modulated p120ctn signaling, providing important insights into lung cancer progression.

## Introduction

Lung cancer remains a serious public health problem worldwide, with the tendency toward metastasis leading to a variety of poor outcomes^1^. Inflammation appears to be a driving force in carcinoma cell metastasis^2^, as clinical and epidemiological studies have suggested a strong association among chronic infection, inflammation, and cancer^1^.

Lumican, a class II small leucine-rich proteoglycan, plays major roles in the organization of extracellular matrix (ECM) and is an important modulator of biological functions including tumor-associated inflammation^3^. Moreover, the overexpression of lumican has been found to affect the growth and invasion inhibition of malignant tumors cells^3^. That said, the roles of lumican in tumors are quite variable. As a substratum, lumican induces the reorganization of actin cytoskeleton, reduces focal adhesions, and suppresses the phosphorylated focal adhesion kinase (pFAK) transduction pathway, and may thus inhibit the migratory phenotype of melanoma cells^4^. In contrast, elevated levels of lumican in extracellular space have been found to result in filamentous actin reorganization and to increase the migration capacity of colon cancer cells^5^. It is thus currently somewhat unclear that what role lumican plays in the invasiveness and metastasis of cancer cells in general.

p120 catenin (p120ctn) is an intracellular scaffolding protein of the catenin family that stabilizes the formation of cadherin-based adhesions and integrates cadherin, Src, and receptor tyrosine kinase signaling through the scaffolding of intracellular signaling molecules^6,7^. p120ctn has a full central Armadillo repeat domain that can interact with the juxtamembrane domain of cadherins in order to participate in the formation of an adhesion complex on the cell membrane^8^. Importantly, p120ctn may regulate the activity of Rho family GTPases through multiple interactions with Rho-GEFs, Rho-GAPs, and their effectors^9^. Small GTPases are involved in the reorganization of microfilament and microtubule network formation that controls cell protrusions such as lamellipodia and filopodia^10^.

In lung cancers, lumican expression occurs in both cancer cells and stromal cells in adenocarcinoma and squamous cell carcinoma, and the expression of lumican in these cells differentially correlates with the clinicopathological findings in such cases.

In this study, we used siRNAs, shRNA, and sgRNAs of lumican approach to analyze the effects of lumican in lung cancer cells. We found that a functional effect of lumican on cancer cell invasion occurs via the physical interaction of tubulin and p120ctn. Functional implications including a role of lumican in p120cn-mediated lung cancer cell invasion are discussed.

## Results

### Depletion of lumican increased metastatic capability

**Fig. 1:**
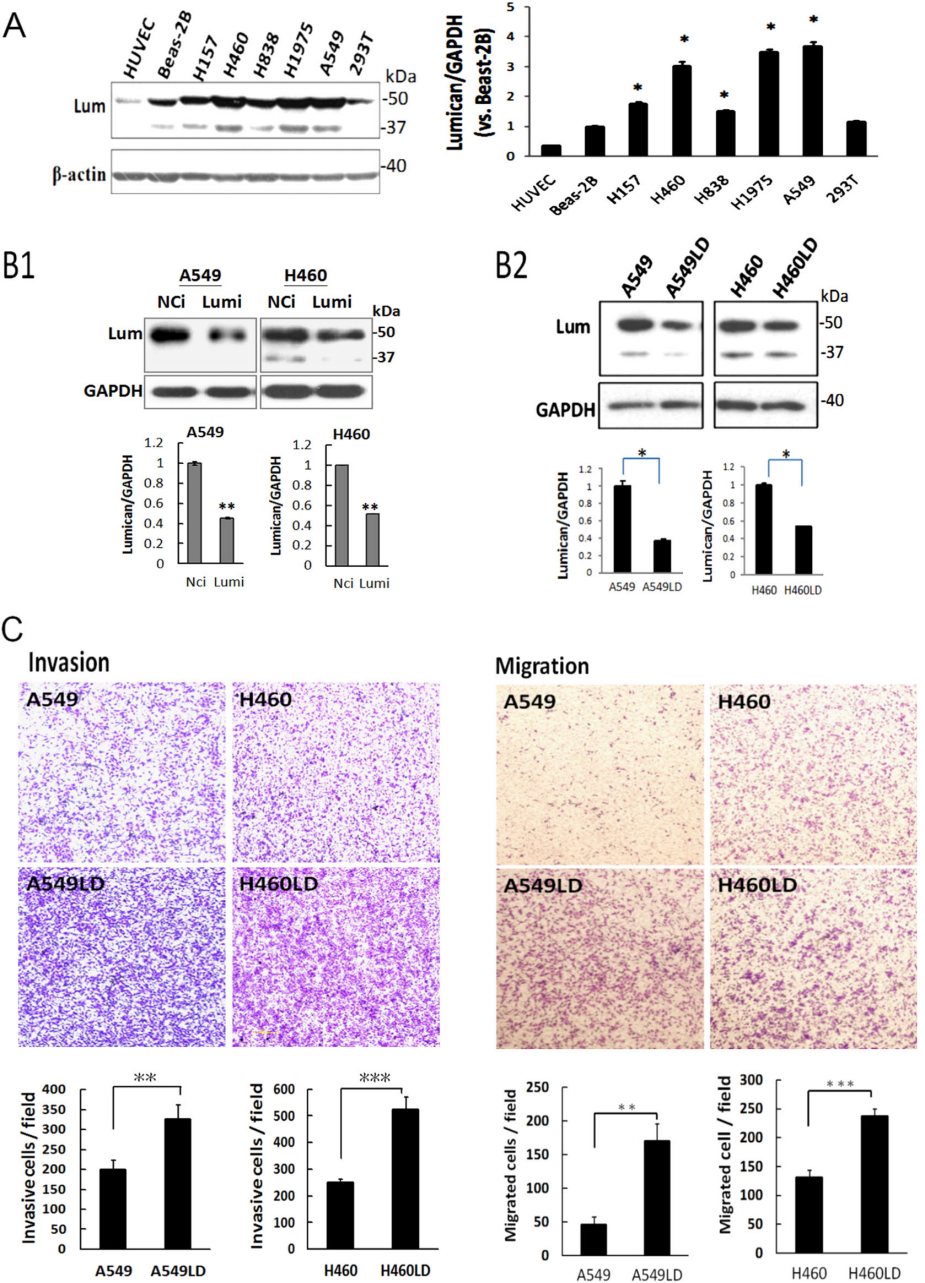
Knockdown of lumican increased metastatic capability. **a** Overexpression of lumican in lung cancer cells. The cell lysates for indicated cells or cell lines were subjected to western blotting for the detection of lumican expressions. **b1** & **b2** Downregulation of lumican with siRNAs or shRNA-specific targeting lumican. B1 A549 and H460 cells were transfected with lumican siRNAs that contained three siRNAs targeting lumican for 24 h and the transfected cells were subjected to western blotting with indicated antibodies. B2 Stable clones for lumican-shRNA-transfected cells. A549 and H460 cells were transfected with lumican shRNA for 24 h and the transfected cells were then selected with puromycin for 4 days. The stable clones were termed as A549LD and H460LD cells. The level of lumican was detected by western blotting. **c** Downregulation of lumican accelerated the cell migration (right panel) and invasion (left panel) capability. A549/A549LD and H460/H460LD cells were seeded in Boyden chambers with or without matrix-gel coating to detect the cell invasion or cell migration. The migrated or invasive cells were counted in five random fields after staining of the cells with crystal violet. The data were expressed as the mean ± SD of three independent experiments. Significantly different from control (*p < 0.05, **p < 0.01,***p<0.001).

Serum lumican levels have been reported to be higher in lung cancer patients as compared to normal controls^11^. In this study, we first examined the lumican expressions in various human cell lines. The overexpression of lumican was found in lung cancer cell lines, but not in human endothelial cells (HUVECs) or transformed lung fibroblasts (Beas-2B) (Fig. 1a). To achieve efficient and specific lumican gene inhibition in lung cancer cells, we used siRNAs and shRNA to approach. The expression level of lumican decreased by 55% and 53% in lumican siRNAs-transfected A549 and H460 cells compared with negative control siRNA (NCi)-transfected cells, respectively (Fig. 1B1). To confirm the specific effect of lumican on lung cancer cells, stable clones were developed by transfecting a lumican shRNA expression plasmid into the A549 and H460 cell lines, and the resulting cell lines were referred as A549LD and H460LD, respectively. western blotting analysis revealed that the downregulation of lumican was exhibited in A549LD and H460LD cells by 55% and 50% compared with A549 and H460 cells, respectively (Fig. 1B2). The data suggested the efficiency of siRNA or shRNA delivery, or the capacity of RNA interference (RNAi) machinery might vary in different cells. The functions of differential expression of lumican in A549/A549LD and H460/H460LD cells were investigated in this study.

The invasion and migration capabilities of the A549LD/A549 and H460LD/H460 cells were investigated by Boyden–Chamber assay with or without matrix-gel coating. Cell invasion increased by 1.63- and 2.10-fold (326.00 ± 38.86 vs. 199.67 ± 24.19 and 525.33 ± 44.99 vs. 249.67 ± 12.50 cells/field, respectively; *p* < 0.05 in both comparisons), and cell migration increased by 3.71- and 1.81-fold (170.67 ± 24.91 vs. 46.00 ± 11.36 and 237.67 ± 12.50 vs. 131.33 ± 13.05 cell/field, respectively; *p* < 0.05 in both comparisons) in the A549LD and H460LD cells compared, respectively, with the A549 and H460 cells (Fig. 1c, d). The data thus indicated a role of lumican in the modulation cell invasion.

### Lumican was implicated in macrophage-conditioned media-induced cell invasion

**Fig. 2:**
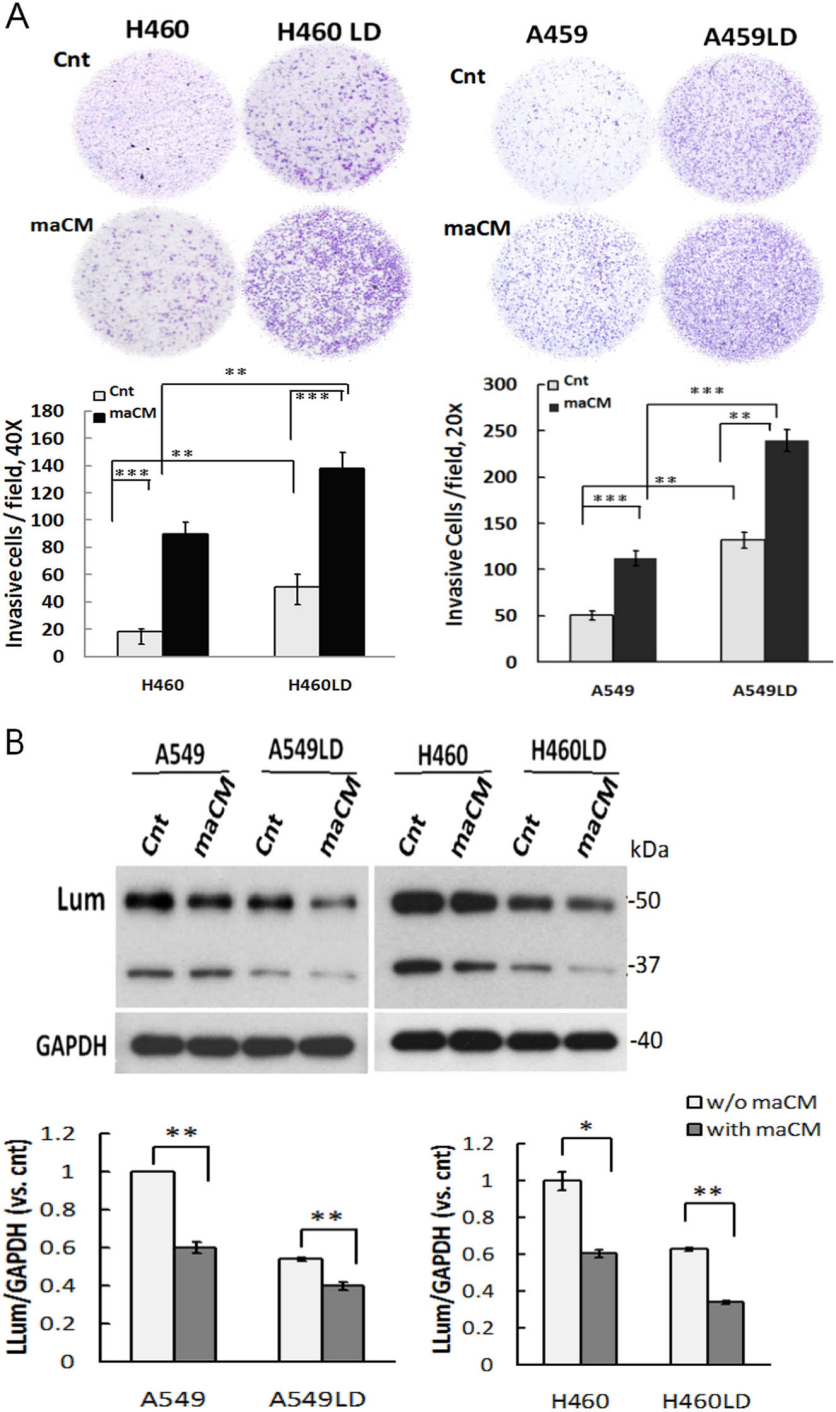
Downregulation of lumican implicated in maCM-induced cancer cell invasion. **a** Downregulation of lumican increased cell invasion and accelerated maCM-mediated cell invasion. The invasion of indicated cells through the membranes of Boyden chambers that were either non-coated or pre-coated with matrix-gel was measured after 24 h incubation at 37 °C. The migrated cells were counted as shown in Fig. 1. **b** Downregulation of lumican associated with maCM-mediated cell invasion. The indicated cell lysates underwent western blot analysis with the indicated antibodies. GAPDH was used as a loading control. Cnt, control medium. Significantly different from control (*p < 0.05, **p < 0.01,***p<0.001).

According to the past studies, infiltrating tumor-associated macrophages were frequently found at the invasive fronts in lung cancers^12^. We further tested the four different types of cells by exposing them to macrophage-conditioned media (maCM). We found that maCM significantly augmented cell invasion in A549, A549LD, H460, and H460LD cells. Furthermore, cell invasion was increased by 2.13- and 1.54-fold overall (240.00 ± 11.79 vs. 112.67 ± 8.50 and 138.00 ± 12.12 vs. 89.67 ± 9.07 cell/field, respectively; *p* < 0.05 in both comparisons) in the A549LD and H460LD cells in comparison to the A549 and H460 cells, respectively (Fig. 2a). By using western blotting, we also found that maCM enhanced cell invasion accompanied by the downregulation of lumican (Fig. 2b). Thus, maCM-accelerated cell invasion might be associated with the modulation of lumican expression.

### Lumican functioned as a tubulin-binding protein and modulated tubulin expression

**Fig. 3:**
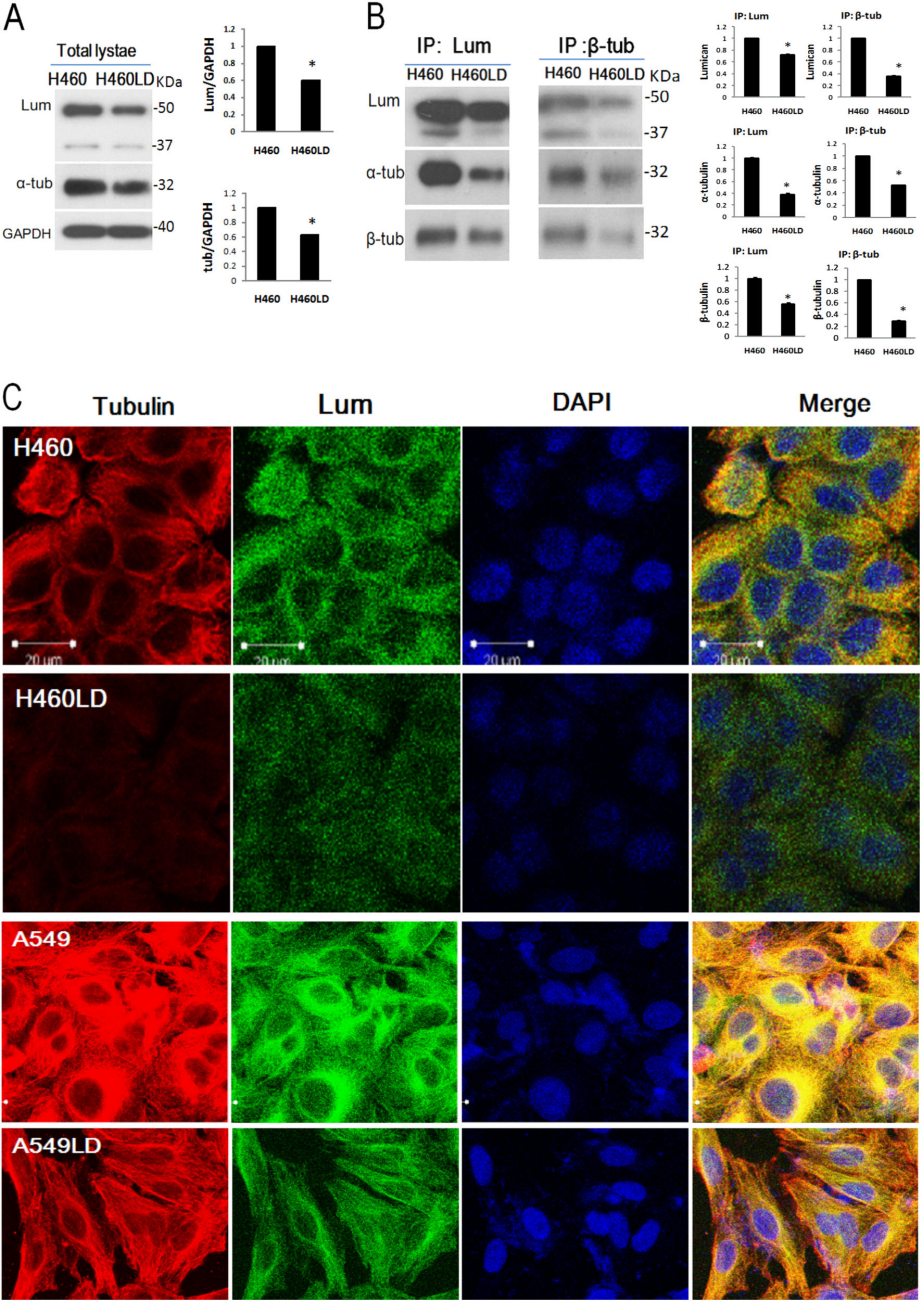
Lumican co-localized with tubulin and regulated tubulin expression. **a** The total cell lysates of H460 and H460LD cells underwent western blot analysis, and the levels of tubulin and lumican were detected. GAPDH was a loading control for the cytosol fraction. **b** Immunoprecipitation detection of lumican and tubulin. Cell lysates were immunoprecipitated with anti-β-tubulin or lumican antibodies. The immunoprecipitates underwent western blot analysis with indicated antibodies. **c** Fluorescence staining of lumican (green) and tubulin (red), in LD cells and control cells. The indicated cells were fixed with paraformaldehyde and co-incubated with anti-tubulin to detect the microtubule expression. Cell nuclei were visualized by DAPI staining (blue). *N* = 3. Significantly different from control (*p < 0.05).

Because the functions of microtubules are so critical for the existence of eukaryotic cell movement, we investigated the possible mechanical effects of lumican on the formation of microtubules. We found a markedly lower expression of tubulin in LD (A549LD & H460LD) cells (Fig. 3a). The immunoprecipitates containing higher level of tubulin corresponded to the higher level of lumican in the H460 cells than in H460LD cells (Fig. 3b), suggesting a physical interaction of lumican and tubulin. The double immunofluoresence staining showed that the expression of α-tubulin (red) in the LD cells was markedly lower than in the control cells (Fig. 3c). Lumican (green) and tubulin were markedly enriched in the control cells and the merged images indicated their co-localization (yellow) in the cytosol (Fig. 3c). These data indicated that lumican plays a role in determining the architecture of microtubules.

### Lumican co-localized with p120ctn and loss of lumican impaired p120ctn expression

**Fig. 4:**
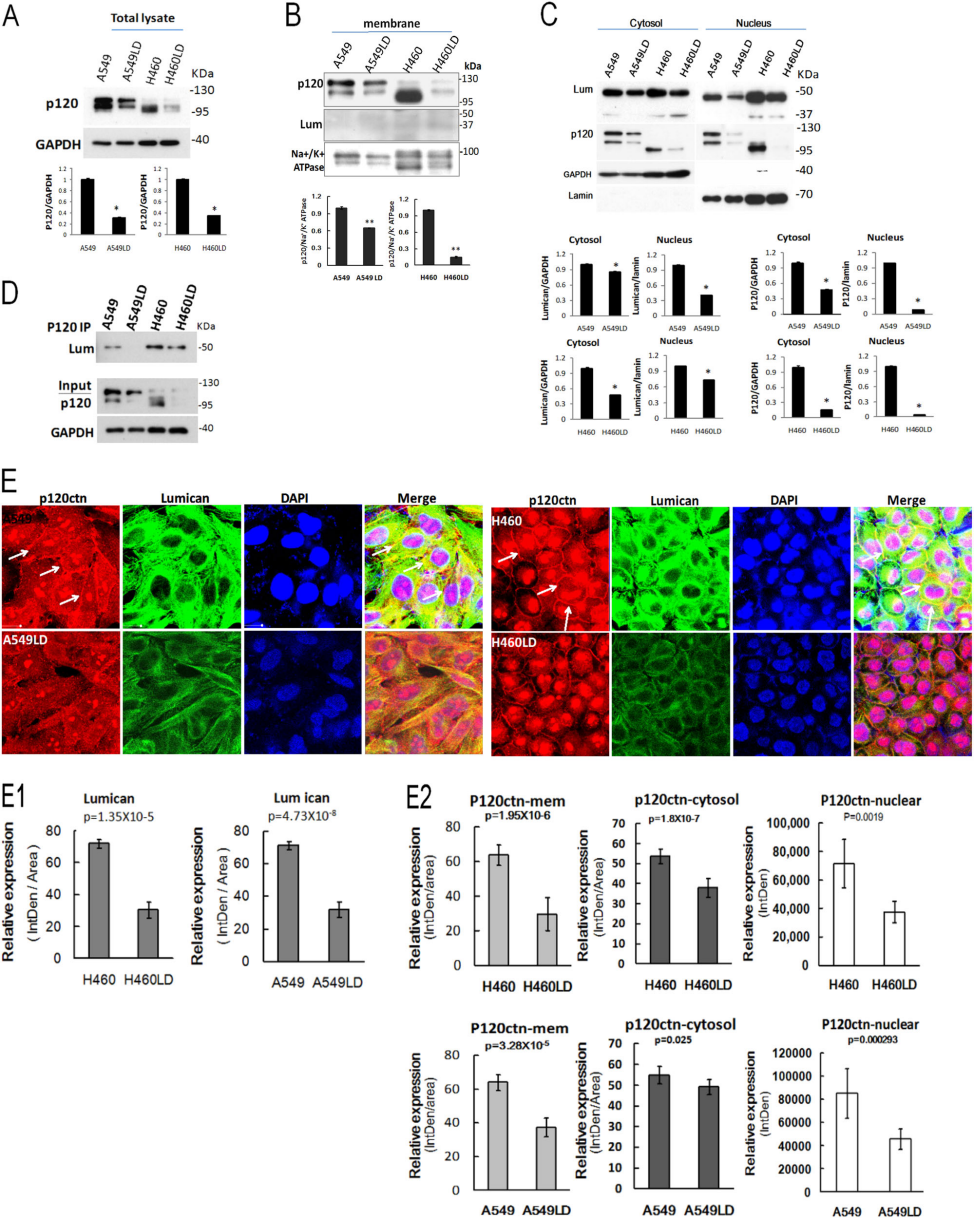
Lumican co-localized with p120ctn and loss of lumican impairs p120ctn expression. The total cell lysates (**a**), the membrane fraction (**b**), and the cytosol/nuclear fractions (**c**) underwent western blot analysis, and the levels of p120ctn and lumican were detected. GAPDH was a loading control for the cytosol fraction. Na^+^/K^+^-ATPase was a loading control for the plasma membrane fraction. **d** Immunoprecipitation detection of lumican and p120ctn. Cell lysates were immunoprecipitated with anti-p120ctn antibody. The total lysate and immunoprecipitates underwent western blot analysis with indicated antibodies. **e** Co-localization of lumican with p120ctn investigated by immunostaining. Cells were fixed with paraformaldehyde and stained with anti-lumican and anti-p120ctn antibodies. Cell nuclei were visualized by DAPI staining (blue). The arrows point out the interactions of p120ctn and lumican at juxtamembrane region. The representative pictures of fluorescent images of lumican (green) and p120ctn (red) were from three independent experiments (E1). The quantitative data of lumican expression were shown in (E2). The fluorescence intensity of p120ctn distribution was shown. Significantly different from control (*p < 0.05, **p < 0.01).

The mechanical effects of this lumican-mediated invasive characteristic were further examined. A basic motif within the p120ctn Arm repeat domain is required for binding to microtubules^13^. The modulation of p120ctn by lumican was firstly investigated. The level of p120ctn was markedly less expressed in the LD cells compared to the control cells (Fig. 4a). The subcellular distributions of p120ctn and lumican were investigated. In the membrane fraction by western blot assay, the level of p120ctn was higher in the control cells than the LD cells and the level of lumican was hardly detected in the control and the LD cells (Fig. 4b). In the cytosol/nuclear fraction by western blot assay, the level of p120ctn and lumican of the LD cells was lower than in the control cells (Fig. 4c). To test whether or not lumican and p120ctn interacted physically, p120ctn was immunoprecipitated from the total cell lysates of the control and LD cells, and then the level of lumican was elucidated by western blot. The immunoprecipitates containing higher levels of lumican were detected in the control cells than the LD cells (Fig. 4d). Furthermore, the possible physical interaction between lumican and p120ctn was elucidated by double immunofluoresence staining analyses (Fig. 4e). The expression of lumican (green) was enriched in the cytosol, and the levels of lumican in the A549LD and H460LD cells were about 55 and 58% lower than those in the A549 and H460 cells, respectively, according to estimations of the immunofluoresence intensities of the cell images (Fig. 4e, the quantitative data were shown in the Fig. 4E1). The expression levels of p120ctn distributed in the membrane, the cytosol, and the nucleus region were significantly decreased in the LD cells (the fluorescence intensity is shown in the 4E2). In contrast, a predominant decrease in the co-localization of lumican and p120ctn was detected at the juxtamembrane region in the LD cells. The data indicated that the formation of lumican-mediated cell–cell contacts was accompanied by the sequestering of p120ctn to the junction regions.

### p120ctn-modulated cell invasion associated with lumican expression

**Fig. 5:**
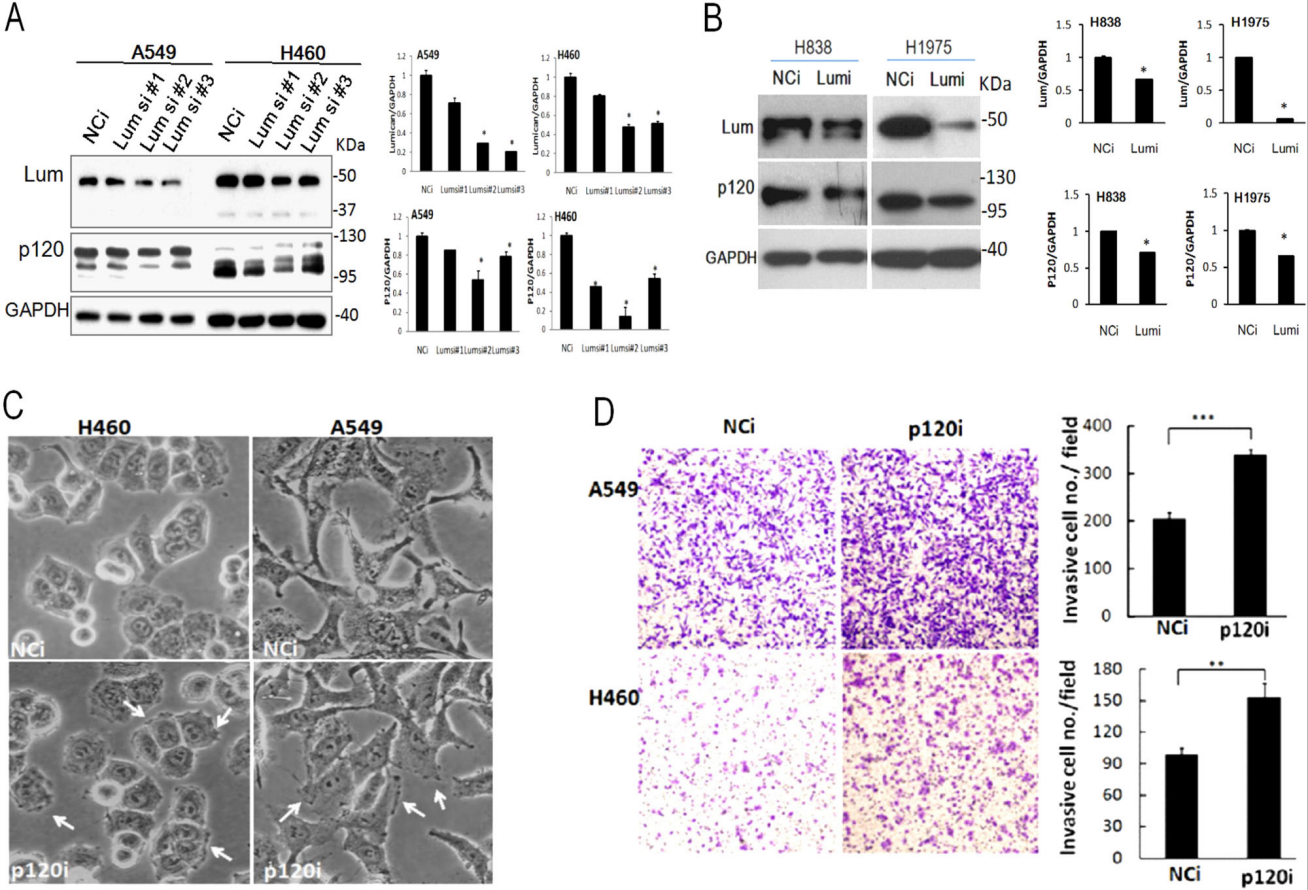
Lumican participated in modulation of cell invasion via p120ctn pathway. **a** Silencing of lumican decreased p120ctn expression. A549/H460 cells were transfected with three types of siRNA designed to knockdown lumican. Cell lysates underwent western blot analysis with the indicated antibodies. **b** Depletion of lumican by lumican siRNA decreased p120ctn expression in H838 and H1957 cell lines. H838/H1957 cells were transfected with lumican siRNA for 24 h. Cell lysates underwent western blot analysis with the indicated antibodies. **c** Micro-observation of p120ctn siRNA-transfected cells. Cells were transfected with p120ctn siRNA for 36 h. The morphologies of the transfected cells were observed and photographs were taken under a phase-contrast microscope. **d** Knockdown of p120ctn increased the cell invasion detected by Boyden–chamber assay with membranes pre-coated with matrix-gel after p120ctn siRNA transfection. The migrated or invasive cells were counted in five random fields after staining of the cells with crystal violet. The data were expressed as the mean ± SD of three independent experiments. Significantly different from control (**p < 0.01, ***p < 0.001). p120i, inhibition of p120ctn by siRNA; NCi, treated with negative control siRNA

The regulation of p120ctn expression by lumican was then verified by transfection with three various lumican siRNAs. The results showed that p120ctn was obviously less expressed in cells subjected to transfection with a variety of lumican siRNAs than in those treated with negative control siRNA (NCi) (Fig. 5a). The effect of lumican on the expression of p120ctn was further confirmed in other NSCLC cell lines, namely, the H838 and H1957 lines. The knockdown of lumican significantly decreased the levels of both lumican and p120ctn expression (Fig. 5b). As is well known, p120ctn plays a major role in cell adhesion and motility through its interactions with Rho^14^. We observed that membrane ruffles and membrane protrusions were more numerous in the p120ctn siRNA (p120i)-transfected cells (Fig. 5c, ruffles indicated by white arrows), about increased by 3.64- and 2.17-folds, respectively (25 ± 2% vs. 91 ± 9% and 46 ± 3% vs. 100 ± 0%, respectively; *p* < 0.05 in both comparisons). Importantly, the cell invasion capability was 1.65- or 1.55-fold (339.00 ± 10.54 vs. 205.67 ± 13.20 or 153.00 ± 13.53 vs. 99.00 ± 6.08 cell/field, respectively; *p* < 0.05 in both comparisons) higher in p120i-transfected cells than in NCi-transfected cells of the A549 or H460 group cells, respectively (Fig. 5d). These data suggested that the depletion of lumican were associated with the downregulation of p120ctn and enhanced cell invasion.

### Knockdown of lumican activated Rho family

**Fig. 6:**
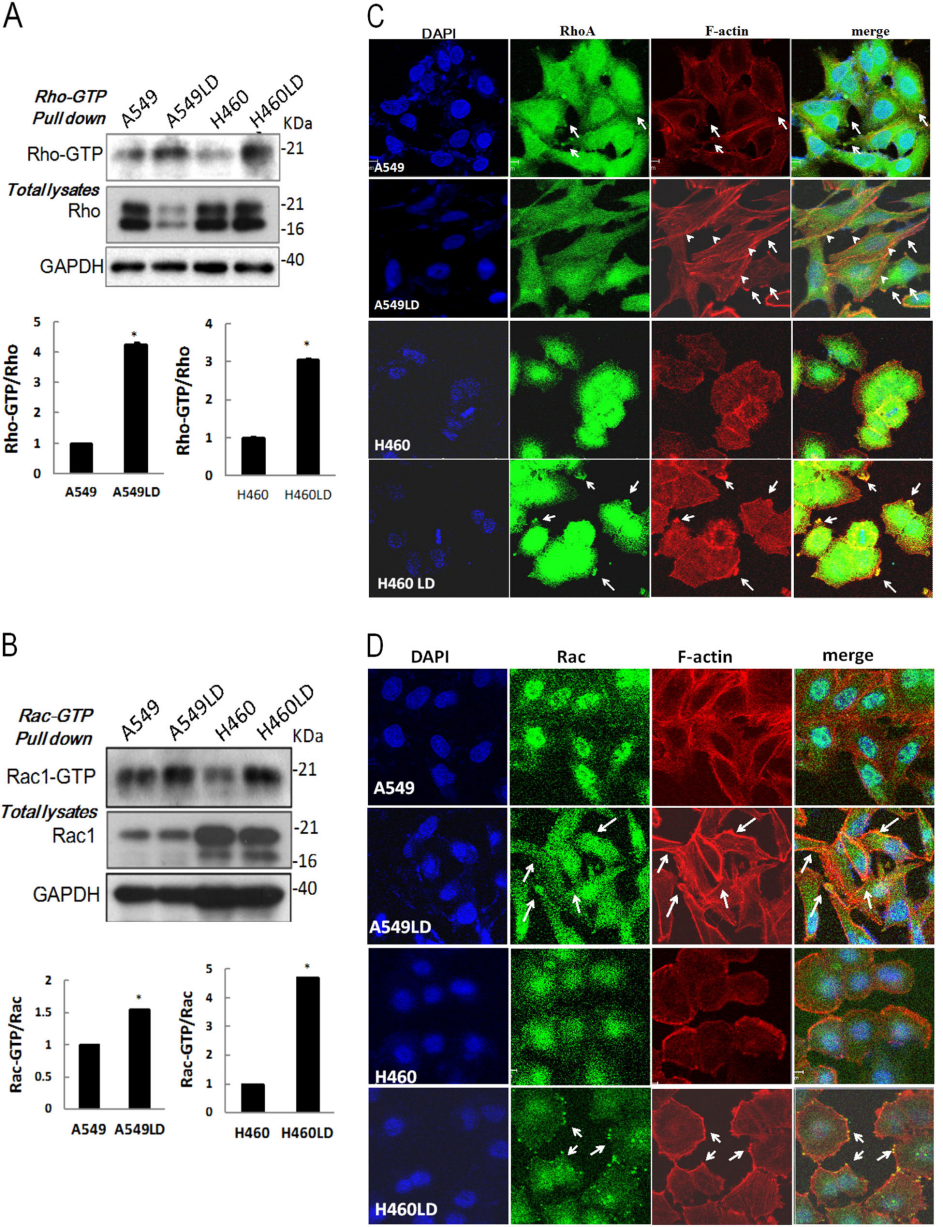
Loss of lumican led to activation of Rho and Rac. **a**, **b** Downregulation of lumican increased the activities of Rho and Rac as determined by pull-down assay. Total cell lysates were subjected to the pull-down assay for Rho and Rac activities. GST-tagged Rhotekin-RBD protein on agarose beads for RhoA or GST-tagged PAK-PBD protein bound agarose beads for Rac were used to bind and precipitate only the active form of RhoA or Rac1 in the cell lysates. The amount of RhoA/Rac1 visualized by immunoblotting represents the amount of GTP that were bound. Total cell lysate was analyzed for RhoA/Rac1 expression as a loading control. Representative blots for GTP-Rho and total Rho are shown in **a**, while those for GTP-Rac and total Rac are shown in **b**. GAPDH was used as the equal protein loading control. The ratio of GTP-RhoA/total Rho (or GTP-Rac/total Rac1) between A549LD and A549 cells, or between H460LD and H460 cells, was analyzed by densitometry of the blot and is expressed in arbitrary units (*N* = 3). The data were expressed as the mean ± SD of three independent experiments. *Significantly different from control (*p < 0.05, **p < 0.01). **c**,** d** Loss of lumican leads to altered RhoA/Rac1 subcellular localization. **c** Immunofluoresence for Rho with F-actin. The cells were fixed and immunostained with anti-RhoA antibody, and then stained with Alexa Fluor 555 phalloidin and with reagent containing DAPI for nucleus. Representative pictures of fluorescent images of RhoA (green) and F-actin (in red) for each genotype from three independent experiments. The nuclei are visualized in blue. The arrowheads point out the stress fibers. The arrows point out the membrane protrusions. **d** Immunofluoresence staining for Rac with F-actin. Cells were fixed with paraformaldehyde and stained with anti-Rac1 antibody and Alexa Fluor 555 phalloidin to detect F-actin expression as shown in **c**. Representative pictures of fluorescent images of Rac1 (green) and F-actin (in red) for each genotype from three independent experiments. The nuclei are visualized in blue. The arrows point out the membrane ruffling. Significantly different from control (*p < 0.05).

p120 regulates cytoskeletal re-organization by affecting the activities of Rho GTPases, or by mediating the association of microtubules to the cell junctions^6^. To assess the responses of Rho and Rac to lumican depletion in lung cancer cells, we performed pull-down assays using GST fusion proteins that bind activated Rac or Rho. As shown in Fig. 5, although the levels of Rho protein were markedly reduced in A549LD and H460LD cells compared to A549 and H460 cells, respectively, the levels of Rho-GTP were 1.7- and 2.6-fold higher in the A549LD and H460LD cells than in the A549 and H460 cells, respectively (Fig. 6a). Similarly, the levels of Rac-GTP were 1.3- and 1.7-fold higher in the A549LD and H460LD cells than in the A549 and H460 cells, respectively (Fig. 6b). The activated Rho/Rac organizing in the actin cytoskeleton was further investigated by immunofluoresence staining. Lung cancer cells were stained with F-actin phalloidin-fluorescin and the antibodies to Rac and Rho. In A549 and H460 cells, phalloidin–fluorescin staining showed a haphazard F-actin arrangement, whereas Rho staining (Fig. 6c) and Rac staining (Fig. 6d) were restricted to the perinuclear region. Extensive membrane protrusions and membrane ruffles indicated F-actin polymerization in the A549LD and H460LD cells, and this polymerization clearly presented at the periphery of the LD cells The prominent thin F-actin microspike-like structures (filopodia) were found to be distributed in these LD cells (Fig. 6c, d). Notably, the merged fluorescence images indicated the co-localization of Rho with stress fiber (shown by arrow heads) in the membrane protrusions in the A549LD and H460LD cells (Fig. 6c, white arrows). The level of Rho co-localization in the membrane ruffle was 3.19- or 6.8-folds higher in the A549LD or H460LD cells than in the A549 or H460 cells, respectively (17.17 ± 4.63% vs. 54.92 ± 0.08% or 7.20 ± 1.49% vs. 49.00 ± 1.00% of cells, respectively; *p* < 0.05 in both comparisons). In contrast, intense co-localization of Rac and polymerized F-actin were obviously observed in the membrane ruffles (Fig. 6d). The level of Rac co-localization in the membrane ruffles was 3.24- or 3.91-folds higher in the A549LD or H460LD cells than in the A549 or H460 cells, respectively (15.84% ± 1.55% vs. 51.19 ± 1.19% or 15.15 ± 1.52% vs. 59.38 ± 9.38% of cells, *p* < 0.05 in both comparisons). The data suggested that the recruitment of these Rho-GTPases to the F-actin structures (membrane protrusions and membrane ruffles) were induced by lumican downregulation.

### Knockdown of lumican activated Rho/LIMK/cofilin pathway

**Fig. 7:**
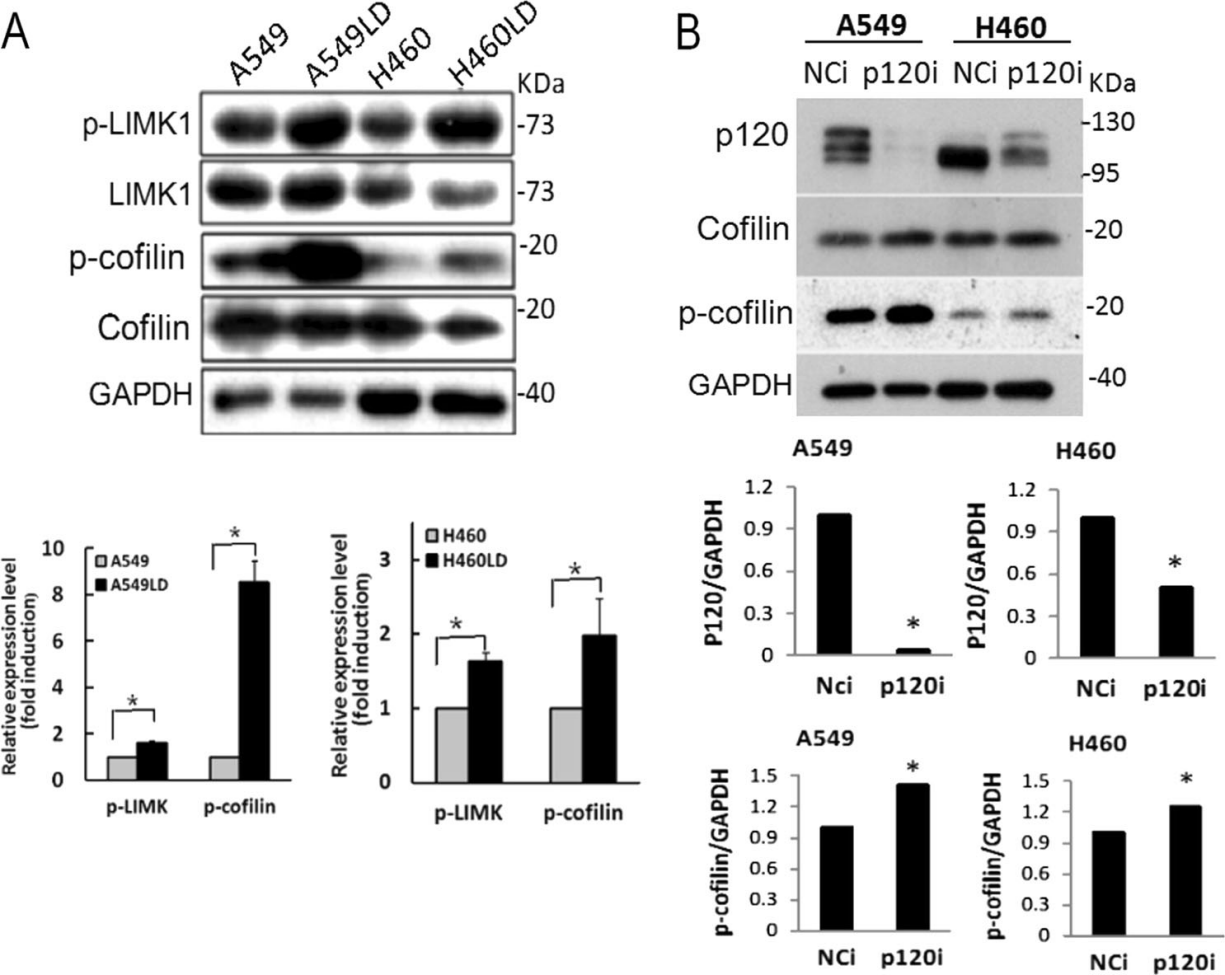
Silencing of lumican activated Rho/LIMK/Cofilin pathway. **a** The cell lysates of cells were subjected to western blotting with the indicated antibodies. The expression levels of p-LIMK or p-cofilin in A549LD and A549 cells, or in H460LD and H460 cells, were determined by densitometry of the blots and are expressed in arbitrary units. **b** Downregulation of p120ctn induced p-cofilin expression. A549 or H460 cells were transfected with p120ctn siRNA for 24 h, and the cell lysates underwent western blot analysis with the indicated antibodies. The data were expressed as the mean ± SD of three independent experiments. Significantly different from control (*p < 0.05).

To investigate whether the activation of the Rho family during the loss-of-lumican triggers the activation of the downstream effectors, the Rho/LIMK (LIM kinase)/cofilin signaling pathway was assessed. The phosphorylated levels of LIMK (p-LIMK) were 1.81- and 1.71-fold and the phosphorylated levels of cofilin (p-cofilin) were 9.1- and 2.09-fold higher in the LD cells than in the control cells, respectively (Fig. 7a). Moreover, the effect of p120ctn on lung cancer cells was assessed by transfection with its specific siRNA. The knockdown of p120ctn efficiently reduced the expression of p120ctn and significantly increased the level of p-cofilin (Fig. 7b). These results indicated the activation of the Rho signaling pathway in the LD cells. Our data might thus indicate that the effects of lumican on the invasiveness of cancer cells were associated with p120ctn-mediated Rho family signaling.

### Knockout of lumican by gRNAs/Cas9 reduced lung cancer cell viability

**Fig. 8:**
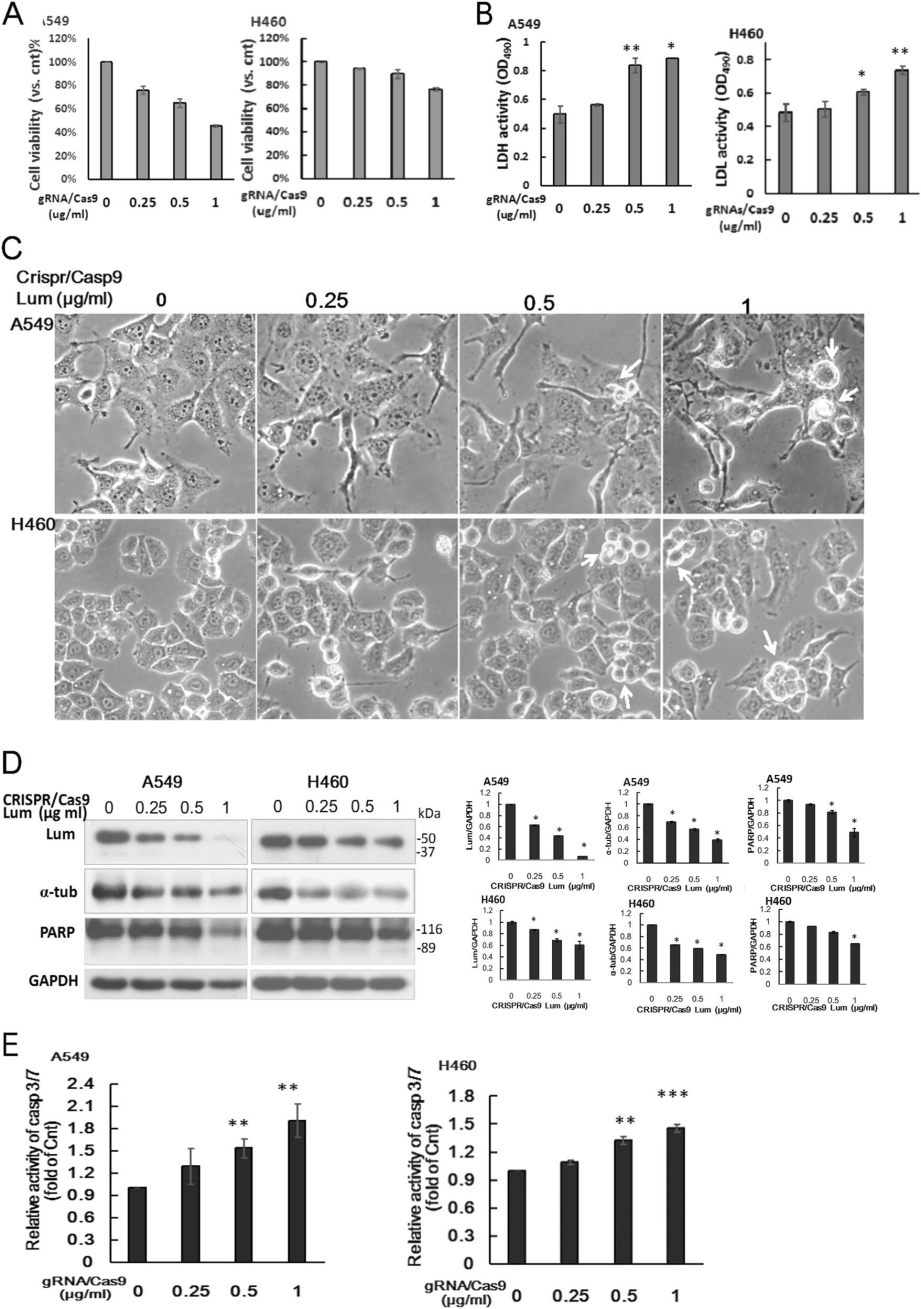
Knockout of lumican by gRNAs/Cas9 induced morphological change and cell death. A549 and H460 cells were transfected with lumican CRISPR/Cas9 KO plasmid (gRNAs/Cas9) (0, 0.25, 0.5, and 1 μg/ml) for 48 h. **a** The cell viability was investigated by the colorimetric MTS assay. **b** The LDH activity in the conditioned media was measured by the CytoTox 96 assay. **c** The cell morphology were shown in higher magnification (10x40) after transfection of Crispr/Casp9 Lum for 48 h. **d** The cell lysates were subjected to western blotting with indicated antibodies. The expressions of lumican, α-tubulin, and full length PARP were determined by densitometry of the blots and expressed in relative arbitrary units. **e** The active caspase-3 was detected by Caspase 3/7-GLO assay. Caspase 3/7 activity of gRNAs/Cas9-untreated control group of each cell line was set as 1. Activities of transfected cells were compared to control group. The data were expressed as the mean ± SD of three independent experiments. Significantly different from control (*p < 0.05, **p < 0.01,***p<0.001).

To determine if knockout of functional lumican gene could specifically change cancer cell function, we introduced lumican gRNAs CRISPR/Cas9 plasmid (0.25 ~ 1 μg/ml) into A549 and H460 cells for 48 h. Obviously, transfection with lumican gRNAs CRISPR/Cas9 plasmids concentration dependently decreased the cell viability (Fig. 8a) and increased the lactate dehydrogenase (LDH) activity in the conditioned media (Fig. 8b). In addition, the morphology of transfected cells changed markedly with filopodia and cell blebbing (white arrow, Fig. 8c). Lumican gRNAs effectively disrupted the expression of lumican, accompanied with decreased expressions of α-tubulin, p120ctn, and poly (ADP-ribose) polymerase (PARP) (Fig. 8d). Strikingly, the knockout of lumican increased the caspase 3/7 activity by 0.2 ~ 1.1 and 0.1 ~ 0.5-fold in A549 and H460 cells, respectively (Fig. 8e). Thus, knockout of lumican might result in caspase-mediated death.

## Discussion

Differential expression of lumican has been shown in normal versus cancer tissues^3^. This study demonstrated the previously unknown roles of lumican and p120ctn in lung cancer cell metastasis. We found that lumican might function as a tubulin-binding protein and contribute to the architecture of microtubules. Interestingly, we also provided the first demonstration of a scaffold interaction between lumican and p120ctn, which might co-localize at the adherent junction. The depletion of lumican-induced microtubule instability and resulted in lung cancer cell invasion via the downregulation of p120ctn. The downregulation of lumican also increased the activities of Rac and Rho, which were implicated in actin cytoskeleton remodeling. Furthermore, the loss-of-lumican decreased the subcellular distribution of p120ctn protein, a finding which indicates a possible mechanism for lumican in lung cancer cell invasion through p120ctn signaling. Through that signaling, the mechanical effects of lumican were implicated in the modulation of microtubule dynamics and p120ctn signaling that governed the lung cancer cell invasion. Moreover, knockout of lumican gene by gRNAs CRISPR/Cas9 decreased the cell viability and induced cell death. Thus, a basal expression of lumican might play a vital role for cancer cell survival.

There are several emerging candidates for receptors and signaling pathways that could mediate lumican’s biological activities and most of these pathways are tissue specific. Surprisingly, we found that the effect of lumican was associated with its tubulin-binding activity. Specifically, the depletion of lumican by shRNA induced the microtubule instability associated with a reduced expression of tubulins and the disruption of microtubule dynamics (Fig. 3). This effect might lead to the loss of the connection between p120ctn and lumican/microtubules (Figs. 4 and  3). Microtubules are one component of the cytoskeleton. They provide platforms for intracellular transport and are involved in a variety of cellular processes, such as microtubule spindle. This study indicated that a scaffold interaction between lumican and tubulin stabilized the p120ctn co-localized at the adherent junction. Thus, our results indicated a previously unknown property of lumican, i.e., it primitively functions as a tubulin-binding protein that tightly controls cell invasion. Knockout of lumican by gRNAs/Cas9 also induced the morphological change (Fig. 8). Lumican gRNAs/Cas9 may be an effective strategy to knockout lumican gene expression, but A549 cells can not survive such a loss-of-gene function (Fig. 8).

This study also revealed the role of lumican-mediated lung cancer cell invasion played via p120ctn. The scaffolding protein p120ctn has critical roles in various cellular signaling pathways, in which it carries multiple-binding partners together to facilitate their concerted interactions and functions^15^. The results of this study indicated a physical interaction between lumican and p120ctn (Fig. 4). Through immunoprecipitation and immunofluoresence images assays, we were able to demonstrate the co-localization of lumican with p120ctn, particularly at the juxtamembrane region (Fig. 4d, e). The reduction of lumican, meanwhile, significantly decreased its bindings at the juxtamembrane region (Fig. 4e). More fascinatingly, we found that the downregulation of lumican decreased the p120ctn expression associated with the decreased distribution of p120ctn in the membrane, cytosol, and nuclear fraction in LD cells (Fig. 4b, c). This effect was further confirmed by the knockdown of lumican with a variety of lumican siRNAs in A549 and H460 cells and in H1975 and H838 lung cancer cells (Fig. 4a, b). The association of p120ctn with lumican/microtubules may lead to their stabilization. It has thus been suggested that p120ctn plays a role in the development and metastasis of cancers, including in human lung cancer cells^8,14^. Consistent with earlier reports, the silencing of p120ctn in A549 and H460 cells in this study also increased the invasion ability of those cells.

It has been shown that p120ctn is an important modulator of the small GTPases that mediate cytoskeletal dynamics^8^. Depending on its localization, p120ctn shows distinct modes of Rho-GTPase regulation. Membrane-associated p120ctn inhibits RhoA activity by binding small-GTPases or interacting with p190RhoGAP, a protein which increases RhoA GTPase activity. In this study, the depletion of lumican decreased the subcellular level of p120ctn in LD cells (Fig. 4). This study also demonstrated that the downregulation of lumican induced stress fiber and membrane ruffle formation effects, which indicated an induction of actin filament remodeling (Fig. 6c, d). Such changes might be associated with the activation of Rho and Rac (Fig. 6a, b) that increased the cells’ motility capabilities (Fig. 1). The downstream target of Rho is mainly involved in the formation of stress fibers and focal adhesions^16^. Interestingly, knockdown of lumican markedly decrease the expression of Rho protein in A549 cells (Fig. 6a). This effect was also detected in H1975 cells after transfecting with lumican siRNA (data not shown). Binding of p120ctn to microtubules is inversely related to its ability to regulate Rho GTPases^17^, but how the regulation of Rho protein by the modulation of lumican is still unclear. In contrast, Rac1 activation might result in actin polymerization and manifest as membrane ruffling at the cellular periphery (Fig. 6d). Such changes provide a highly flexible membrane and a dynamic cytoskeleton and are crucial for rapid cell invasion. p120ctn has been shown to play a mutually different role in regulating Rac1 and Rho A activation^18^. This study indicated that the depletion of lumican induced the activation of RhoA and Rac, suggesting that the other effect of lumican may activate Rac. That said, this mechanical regulation still requires further investigation.

Cofilin has been shown to be a potent regulator of actin filament dynamics, and its ability to bind and depolymerize actin is abolished by phosphorylation of serine residue at three^19^. LIMK is responsible for this phosphorylation and induces actin reorganization and reverses cofilin-induced actin depolymerization^19^. This study indicated that the knockdown of lumican increased the level of p-LIMK by accompanying with the increased p-cofilin (Fig. 7a). It has been shown that p120ctn regulates the actin cytoskeleton via Rho family GTPases^20^. However, increases in p-cofilin seemed to be more pronounced with relatively modest lumican knockdown compared to that seen with more complete p120ctn knockdown (Fig. 7a, b). It is probable that knockdown of lumican might induce alternative signaling(s) to activate the Rho-cofilin pathway. It has been shown that alterations in integrin expression profiles allow cells to modulate the motile machinery through Rho GTPases^21^. Lumican functioned as a tubulin-binding protein. Knockdown of lumican did not only modulate p120ct expression (Fig. 4), but also induced the subtype switch of integrin (Supplementary Fig. 1). It is possible that differences in the extent of integrin clustering have an impact on the intensity of Rho/cofilin signaling. Thus, LIMKs phosphorylated and inactivated the actin depolymerizing factor cofilin may result in a net increase in the cellular filamentous actin that induce cell morphology change.

The results of this study indicated that lumican functions as a tubulin/p120ctn-binding protein. Lumican might affect the organization of p120ctn-based adhesions (i.e., scaffolding) and, in turn, the blocking of p120ctn-dependent signaling. The invasive effect of lumican and p120ctn may be correlated with RhoA and Rac1 signaling. The underlying mechanism of the effect of lumican on p120ctn and adherent junction-related proteins was associated with its architectural role in the production of microtubules. Moreover, the downregulation of lumican was implicated in maCM-mediated invasion with a potential role in tumor-associated inflammatory response (Fig. 2).The potential role of lumican in response to inflammation is needed to be investigated. This study indicated the complex of lumican/microtubule may act as a molecular switch to orchestrate the balance between cellular adhesion and migration, an effect which may, in part, explain its roles in regulating p120ctn signaling and cytoskeletal remodeling.

## Material and methods

### Cell culture

The NSCLC cell lines A549 (ATCC CCL-185), H460 (ATCC HTB-177), H1975 (ATCC CRL-5908), H157 (ATCC CRL-5802), and H838 (ATCC CRL-5844), as well as human umbilical venous endothelial cells (HUVEC), Beas-2B transformed bronchial epithelial cells (ATCC CRL-9609), and transformed human embryonic kidney cells 293T (ATCC CRL-3216), were purchased from the American Type Culture Collection (ATCC; Manassas, Virginia, United States).

### Reagents and antibodies

Unless otherwise indicated, all chemical reagents were purchased from Sigma-Aldrich. Antibody to lumican (ab168348) was purchased from Abcam. Antibodies to RhoA (#2117) and Rac1/2/3 (#2465) were purchased from Cell Signaling Technology Inc. Antibody to p120ctn (#CM3541) was purchased from ECM Biosciences.

### Generation of the stable A549LD and H460LD cell lines with low levels of lumican and CRIPSR/Casp9 lumican knockout

Stable cell lines expressing lower levels of lumican were created by using short hairpin RNA (lumican shRNA plasmid purchased from Santa Cruz, sc-43901-SH) directed against lumican. The transfected A549 or H460 cells were selected by puromycin at 1.0 ìg/mL of medium starting 24 h after transfection. Messenger RNA and protein levels of lumican were measured by reverse transcription-PCR (RT-PCR) or western blotting. The resulting stable cell lines, which were designated as A549LD and H460LD, were separately maintained in culture medium containing puromycin. Specific small interfering RNAs (siRNAs) were then used to silence lumican and p120ctn expression. A variety of siRNAs targeting parts of the lumican mRNA were selected and synthesized by Bio-tool Research Inc. and were identified as follows: Lumican-siRNA#1: 5′-GGGCAAUCAUCACCAAACUTT-3′; Lumican-siRNA#2: 5′-GCCUCCUGGAAUCAAGUAUTT-3′; and Lumican-siRNA#3: 5′-CCACCGGAUAUGUAUGAAUTT-3′. p120ctn-specific siRNA was synthesized from p120ctn siRNA 5′-GCUAUGAUGACCUGGAUUA-3′ and 5′-CUAUGAUGACCUGGAUUAU-3′^14^. Lumican CRISPR/Cas9 KO plasmid (h) is designed to disrupt gene expression by causing a double-strand break (DSB) in a 5′ constitutive exon within the lumican (human) gene (purchased from Santa Cruz, sc-402001). This plasmid (h) consists of a pool of three plasmids, each encoding the Cas9 nuclease and the target-specific 20 nt guide RNAs (gRNAs) designed for maximum knockout efficiency. The cells 1.5 × 10^5^ and −2.5 × 10^5^ were required for CRISPR/Cas9 KO plasmid transfection.

### Collection of conditioned media

THP1-derived macrophages were generated as described in previous studies^22,23^.

### MTS assay, LDH activity and luminescence-based caspase-3/7 activity assay

The cell viability was detected by the colorimetric MTS assay using the CellTiter 96 Aqueous One Solution proliferation assay system (Promega, Madison, WI). LDH, a stable cytosolic enzyme that is released upon cell lysis was measured by the CytoTox 96 non-radioactive cytotoxicity assay (Promega, Madison, WI). Active Caspase 3 was assay with the caspase 3/7-GLO assay (Promega, Madison, WI).

### Matrigel invasion/migration assay

Following the manufacturer’s instructions, in the upper chambers, 2 ~ 5 × 10^5^ cells were grown in serum-free medium on 8 µm porous polycarbonate membranes (Corning, Acton, MA, USA) that were coated with/without Matrigel basement membrane matrix (BD Biosciences). The lower chambers were filled with RPMI 1640 medium containing 10% fetal calf serum. After incubation for 16 h at 37 °C in a humid atmosphere of 5% CO_2_ and 95% air, the cells that had migrated through the pores were fixed with paraformaldehyde for 30 min and stained with crystal violet (Sigma). Then the number of cells was counted visually using a microscope in five different fields under ×200 magnifications per filter. Each experiment was performed in triplicate.

### Immunoprecipitation (IP) and western blot

The cells were lysed in cold lysis buffer (1% NP-40, 50 mM Tris, pH 7.4, 150 mM NaCl, 2 mM EDTA, 50 mM NaF, 10% glycerol, the Halt protease, and phosphatase inhibitor cocktail). The protein concentration was determined using the Bio-Rad protein assay kit. After clearing the lysate with an appropriate pre-immune serum and protein G (Roche), IP was performed using the indicated antibodies. The immunoprecipitates or cell lysates were resolved on SDS-polyacrylamide gel electrophoresis. On blotting the gel to polyvinylidene difluoride membrane, the proteins were detected by appropriate antibodies and visualized by chemiluminescence.

### RhoA or Rac1 activation assay

RhoA or Rac1 activities were measured using a pull-down assay (Rho or Rac activation kit ADI-EKS-465 and ADI-EKS-450, Enzo Life Sciences). The Active Rho and Rac1 pull-down and detection kit was validated for function and specificity for active Rho (Rac1) using cell lysates treated with GTPγS to activate endogenous Rho (Rac) and compared to GDP-treated lysates to inactivate the small GTPase. A549LD and H460LD cells or the cells that were transfected with the p120ctn siRNA were washed in ice-cold PBS and lysed. Equal amounts of whole-cell lysates were incubated with 20 mg of Rhotekin-RBD (Rho Binding Domain of Rhotekin) or PAK-PBD (p21 Binding Domain of p21 Activated Kinase 1) beads for 1 h at 4 °C. The beads were washed three times with washing buffer, and the bound RhoA or Rac1 proteins were analyzed by western blots using an anti-RhoA or anti-Rac1 antibody.

### Confocal immunofluorescence microscope

A549/A549LD and H460/H460LD cells or p120ctn siRNA-transfected cells were grown in chamber-slide glass and incubated for 24 h. The cells were then fixed in 4% formaldehyde at room temperature. After being rinsed in PBS, the cells were incubated overnight with anti-Rac, anti-Rho, or anti-p120ctn, or anti-tubulin at 4 °C. Samples were then incubated with the corresponding Alexa Fluor-488 or -546-conjugated secondary antibodies (Invitrogen-Molecular Probes or Life Technology) for 1 h followed by incubation with Alexa Fluor 555 phalloidin (Invitrogen) or 4′,6-diamidino-2-phenylindole (DAPI, Invitrogen, 1:1000) for 30 min. Samples were visualized using a ZEISS LSM 510 META/Confocor2 microscope (Carl Zeiss MicroImaging) with the pinhole set at one Airy unit using 488, 546, and/or 633 nm excitation and a 63/1.4 oil objective lens. The fluorescence intensity of target proteins was quantified by MetaMorph Microscopy Automation and Image Analysis Software (Molecular Devices, Sunnyvale, CA, USA). Fluorescence density (FD) was defined as each cell fluorescence intensity divided by each cell area (fluorescence intensity/µm^2^). The mean FD of 50–80 different cells in 5 ~ 8 fields at 630× was used to represent the immunofluorescence of target proteins. The proportion of inner-region lumican was determined by comparing the intensity in the inner region to the total intensity in each cell.

### Statistical analysis

The data were presented as the mean ± SD. Statistical significance between groups was assessed by unpaired Student’s *t*-test. The *p*-value of <0.05 was considered to be significant.

## Electronic supplementary material

Supplement Fig. 1(JPG 54 kb)

Supplementary Figure Legend(DOCX 14 kb)
