# Supplementary figures and images for: Downregulation of lumican accelerates lung cancer cell invasion through p120 catenin
Source: Cell Death Dis. 2018 Mar 16;9(4):414. doi: 10.1038/s41419-017-0212-3 (PMC5856799; doi:10.1038/s41419-017-0212-3)

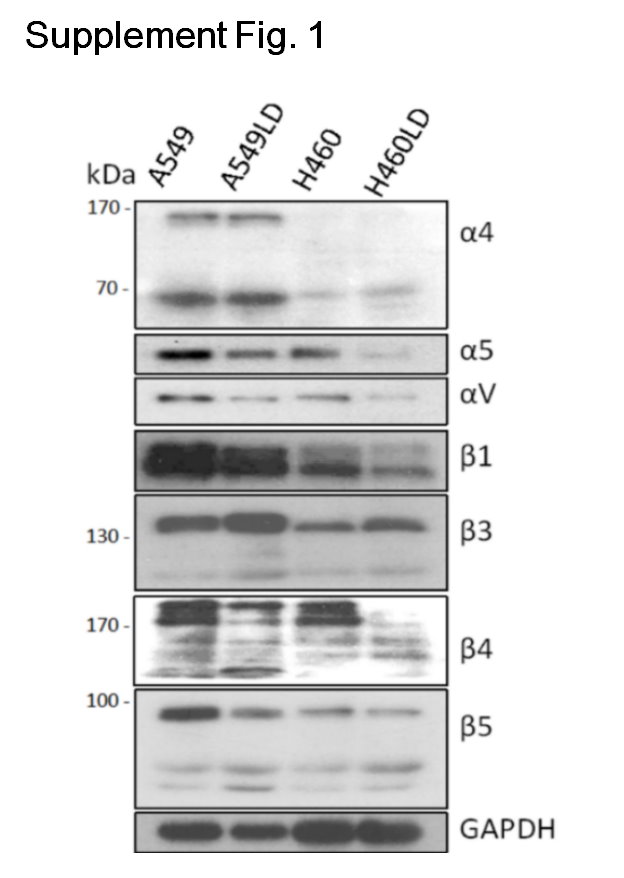

Supplement: Supplementary file 1 — Supplement Fig. 1(JPG 54 kb) [file 41419_2017_212_MOESM1_ESM.jpg]
