# Supplementary material for: Downregulation of lumican accelerates lung cancer cell invasion through p120 catenin
Source: Cell Death Dis. 2018 Mar 16;9(4):414. doi: 10.1038/s41419-017-0212-3 (PMC5856799; doi:10.1038/s41419-017-0212-3)
Supplement: Supplementary file 2 — Supplementary Figure Legend(DOCX 14 kb) [file 41419_2017_212_MOESM2_ESM.docx]

**Supplementary Figure Legend**

**Supplement Fig. 1**. Knockdown of lumican induced the subtype switch of integrin. Cell lysates underwent western blot analysis with the indicated antibodies. The integrin (In) switch was found to be associated with decreased Inα5, αV, β1, β4, and β5 expressions as well as increased expressions of Inβ3, and α4. The data suggested that the effects of lumican on the modulation of cell morphology were associated with changes in focal adhesions. N=3.
